# Bioinformatics approaches to analyzing CRISPR screen data: from dropout screens to single-cell CRISPR screens

**Published:** 2022-12

**Authors:** Yueshan Zhao, Min Zhang, Da Yang

**Affiliations:** 1 Center for Pharmacogenetics, Department of Pharmaceutical Sciences, University of Pittsburgh, Pittsburgh PA 15261, USA; 2 UPMC Hillman Cancer Institute, University of Pittsburgh, Pittsburgh, PA 15261, USA; 3 Department of Computational and Systems Biology, University of Pittsburgh, Pittsburgh, PA 15261, USA

**Keywords:** CRISPR/Cas9, dropout screen, sorting-based screen, single-cell CRISPR screen, drug-gene interaction

## Abstract

**Background:**

Pooled CRISPR screen is a promising tool in drug targets or essential genes identification with the utilization of three different systems including CRISPR knockout (CRISPRko), CRISPR interference (CRISPRi) and CRISPR activation (CRISPRa). Aside from continuous improvements in technology, more and more bioinformatics methods have been developed to analyze the data obtained by CRISPR screens which facilitate better understanding of physiological effects.

**Results:**

Here, we provide an overview on the application of CRISPR screens and bioinformatics approaches to analyzing different types of CRISPR screen data. We also discuss mechanisms and underlying challenges for the analysis of dropout screens, sorting-based screens and single-cell screens.

**Conclusion:**

Different analysis approaches should be chosen based on the design of screens. This review will help community to better design novel algorithms and provide suggestions for wet-lab researchers to choose from different analysis methods.

## Author summary

CRISPR screen has become a promising tool in the identification of essential genes or drug targets. In this review, we provide an overview on the application of CRISPR screens and bioinformatics approaches to analyzing different types of CRISPR screen data. We also discuss mechanisms and underlying challenges for the analysis of dropout screens, sorting-based screens and single-cell screens.

## INTRODUCTION

Clustered regularly interspaced palindromic repeats (CRISPR) loci with endonuclease (Cas) proteins is an immune defense system in bacteria, among which CRISPR-Cas9 is the most common one [1]. Winning the Nobel Prize in 2020, CRISPR technology has become an effective tool for biological research, but its application is more than gene editing. Numerous studies have used CRISPR technology to perform high throughput genome-scale screening and identified essential genes or therapeutic targets.

**Figure 1.**
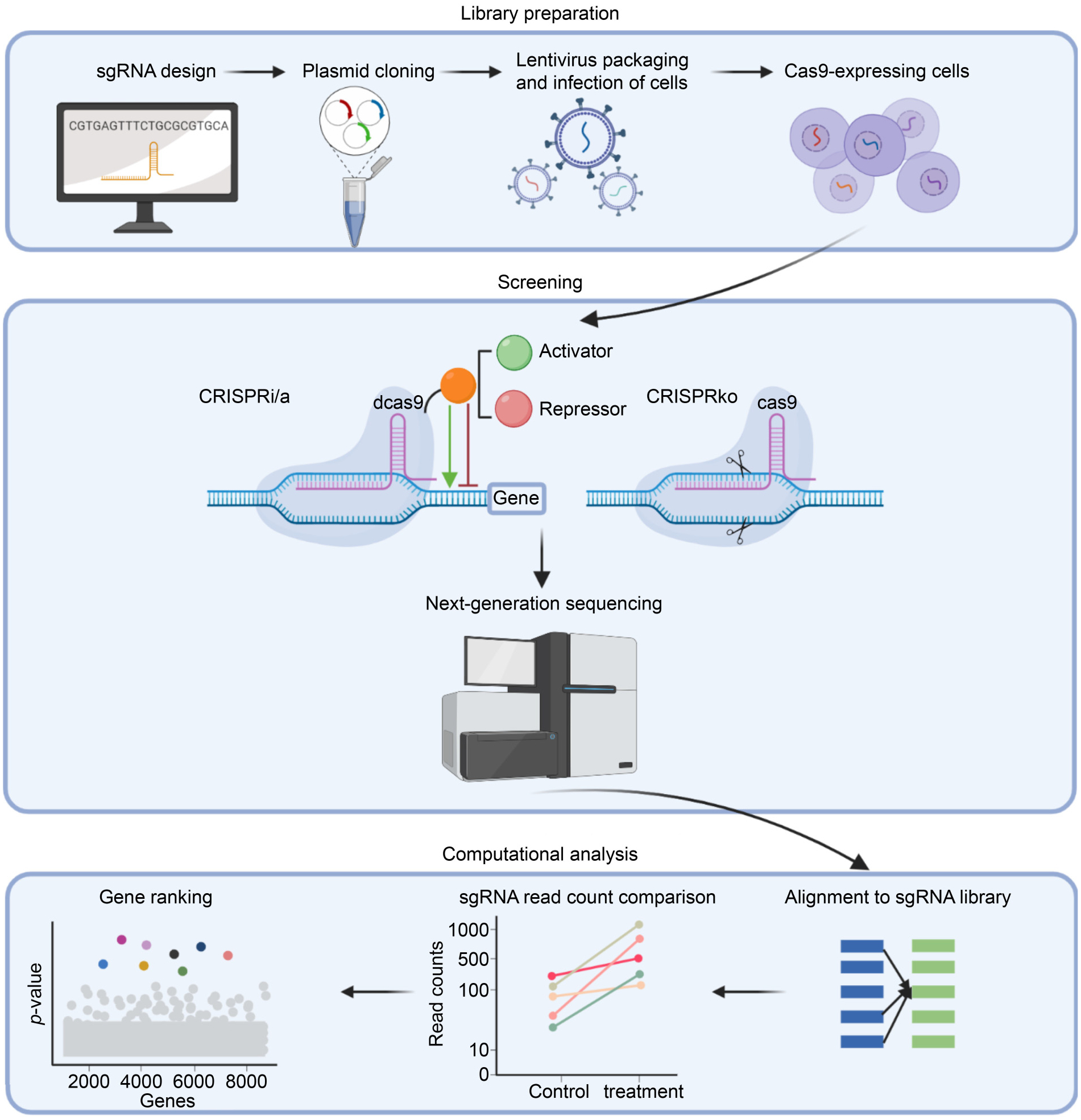
General process for pooled CRISPR screens. Library preparation: Multiple single guide RNAs (sgRNAs) are designed for one target, which can be cloned into plasmids. The lentiviral library is then packaged and used to infect desired cells for CRISPR screens. Screening: After pooled library preparation, targets are edited by either CRISPR knockout or CRISPR interference/activation. Next-generation sequencing is performed to collect sgRNA abundance in cell population. Computational analysis: Following deep sequencing, reads are mapped to the original sgRNA library, and fold changes of sgRNA read counts are then calculated. Based on various algorithms, hit genes can be identified.

The aim of genome-scale screens is to generate a population of cells with different perturbations to identify genes or regulatory regions that will play a role in specific phenotypes. Because of the wide range of potential target sequences, CRISPR system has enabled powerful pooled screens. Based on different mechanisms, CRISPR screens can be categorized into three types: CRISPR/Cas9 knockout (CRISPRko) screens, CRISPR/dCas9 activation (CRISPRa) screens and CRISPR/dCas9 interference (CRISPRi) screens (Fig. 1). In CRISPR knockout screens, Cas9-guided DNA double strand breaks lead to insertions or deletions when cells try to repair DNA with the non-homologous end-joining (NHEJ) pathway [2]. These mutations will result in a coding frameshift or stop codon, which ultimately silence gene expression. The deactivated Cas9 (dCas9) is a mutant protein which is not able to cleave DNA. Fused with transcription activators or repressors, CRISPR-dCas9 system allows regulation at gene transcription level or epigenetic level for either gene activation (CRISPRa) or interference (CRISPRi) [3]. Inhibition of gene expression can be accomplished by fusing transcription repressor such as KRAB (Kruppel-associated box) to dCas9 protein in CRISPRi screens [4]. Similarly, CRISPRa screens take advantage of transcription activator such as synergistic activation mediator (SAM) system [5], which consists of four copies of VP16 from herpes simplex virus and sgRNA containing MS2-p65-HSF1 (heat shock transcription factor 1) domains. Besides protein coding genes screening, CRISPRi and CRISPRa screens can be used for functional characterization of regulatory elements [6] and long noncoding RNAs (lncRNAs) [7]. CRISPRko and CRISPRi facilitate loss of function while CRISPRa allows gain of function. When focusing on gene depletion, CRISPRko is usually preferred because of clearer signals [8].

**Figure 2.**
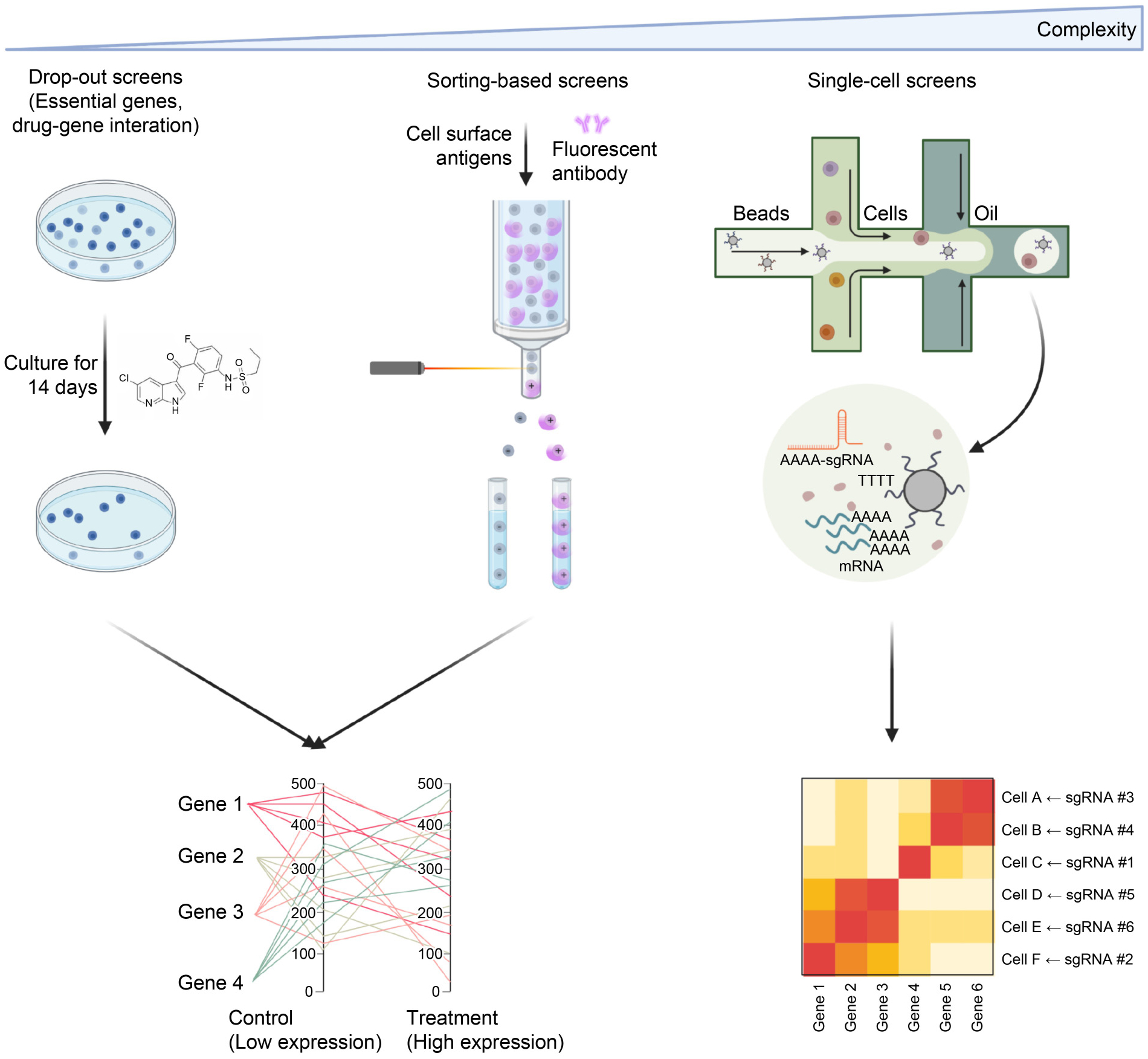
Different phenotypes for CRISPR screens and data analysis. Cell survival is the phenotype used in drop-out screens and cellular libraries are cultured for a period of time (with drug treatment) for identification of essential genes or potential genes conferring drug sensitivity/resistance. Marked by fluorescent antibody, cell surface antigens can be the phenotype used in sorting-based screens. Based on the expression level of certain markers, cells will be sorted into different groups, and sgRNA abundance in high expression group and low expression group will be compared to identify target genes. Further combined with single-cell RNA-seq, effect of gene perturbations on the whole transcriptome can be discovered.

The pooled CRISPR screens were initially used to identify essential genes for cell viability [9]. Combining with fluorescence-activated cell sorting (FACS), the phenotype was extended to cell surface markers [10], intracellular reporters [11] or specific cell types [12]. Further utilization of single-cell RNA-seq (scRNA-seq) of the CRISPR screened samples dramatically expand the dimensions of phenotypes to the expression levels of hundreds of genes simultaneously (Fig. 2). CRISPR screens at single cell level facilitate insights into the effect of gene perturbations on the whole transcriptome, and different methodologies such as Perturb-seq [13,14], CRISP-seq [15] and CROP-seq [16] have been developed.

As a genome wide high-throughput screening technology, whether CRISPR screens can effectively provide insights for us largely depends on the accuracy of data analysis. There have been quite some challenges for the development of CRISPR screen analysis methods. Because of next-generation-sequencing (NGS), we have to handle large size of sequencing data with noise. Meanwhile, due to the fact that multiple sgRNAs are designed for one target, we are also faced with variable sgRNA efficiency and off-target effects. The method is also expected to deal with different phenotype effects from simple cell viability to complicated transcriptome profiles. Despite the difficulty, various methods with different focus have been developed for CRISPR screen analysis. The overall workflow of those methods usually includes sequence quality assessment, read alignment, read count normalization, estimate changes of sgRNA abundance and aggregating sgRNA effects for the overall effects of targeted genes. In addition to those novel algorithms, some previously designed methods for RNA interference (RNAi) screening analysis can be repurposed for CRISPR screens analysis. Here, we will start with a comprehensive review and discussion of those computational approaches specifically developed for CRISPR screens. Next, we will introduce a group of the shRNA screening methods that have been repurposed for CRISPR screens analysis. Finally, we will review the computational platform that can be used for single cell CRISPR screens and drug-gene interaction. A summary of the tools for CRISPR screen data analysis is shown in Table 1.

## METHODS DESIGNED FOR CRISPR SCREENS

### Model-based analysis of genome-wide CRISPR/Cas9 knockout (MAGeCK)

MAGeCK [17] was the first workflow designed for CRISPR/Cas9 screen analysis and has been widely used ever since. The assumption was that if a gene is essential, then sgRNAs targeting this gene will be enriched at one side instead of being randomly distributed in the rank. Read counts in different groups were first normalized for the adjustment of library sizes and count distributions. The sgRNA abundance was over-dispersed like other high-throughput sequencing experiments [18], thus a mean-variance model was utilized to estimate the variance. A negative binomial distribution similar to edgeR [19] package was then used to test whether there is significant difference between treatment and control groups. They further used *p*-values calculated from the negative binomial distributions to rank sgRNAs, and a robust ranking aggregation (RRA) [20] method to identify positively and negatively enriched genes simultaneously. Finally, FDR can be calculated from permutation tests. MAGeCK was also able to identify essential pathways based on the same principle.

Recently, MAGeCK have been further developed into integrated workflows, MAGeCK-VISPR [21] (visualization for CRISPR) and MAGeCKFlute [22], which were able to provide extensive quality control at the sequence level, read count level and sample level. VISPR also provided multiple ways to explore screen results in depth including Gene Ontology (GO) enrichment analysis from GOrilla [23], gene-gene interaction network from GeneMANIA [24] and so on. Although it requires command lines for operation, detailed manuals and instruction videos are available. The MAGeCK series is also constantly updated, and it has become the standard solution for CRISPR screen analysis.

### Screening Bayesian evaluation and analysis method (ScreenBEAM)

A limitation with existing analysis approaches such as MAGeCK is that they have to accurately estimate individual shRNA/sgRNA effects, but it is hard to achieve in reality with the lack of enough replicates. Instead of previous two-step analysis, ScreenBEAM [25] was developed to directly estimate gene-level effects in 2016. They used the linear model equivalent to student’s *t*-statistics to test the significance of coefficient. A Bayesian hierarchical model was introduced, whose parameters estimated the effect of shRNA/sgRNA group. In order to robustly estimate the parameters, Markov Chain Monte Carlo (MCMC) simulations were used. This model can be understood as a mixture linear model that incorporates gene activity and variable guide silencing efficiency. ScreenBEAM outperformed other approaches especially with relatively low-quality screen data, which is small in size with noise. In addition, ScreenBEAM can deal with data obtained from both microarray and large-scale NGS.

### Bayesian analysis of gene essentiaLity (BAGEL)

BAGEL was developed in 2016 for analyzing gene knockout screens [26]. Based on Bayes theorem, BAGEL used predefined essential and nonessential genes from reference gene sets. The sgRNA fold change distribution in both essential and nonessential gene sets was first estimated by a kernel density estimate function. Then, BAGEL evaluated the probability that the abundance changes of all sgRNAs for one gene were extracted from the essential gene distribution or nonessential gene distribution, and a Bayes Factor (BF) was the output result. Bootstrap resampling in the gene sets was further performed and the posterior distribution of BF was reported to determine whether this gene was essential or not. An improved version, BAGEL2 [27], uses a linear regression of log likelihood ratio for score interpolation while BAGEL builds a truncated fold change model to estimate Bayes Factors. Moreover, BAGEL2 offers a 10-fold cross-validation option that works faster. The BAGEL algorithm was able to detect subtle changes in overall gene effect, while algorithms based on null hypothesis such as MAGeCK required deeper sampling or a more obvious phenotype. In negative selection, genes stand out by depletion, so it is optimized to use BAGEL because it incorporates prior knowledge of genes to increase the signal-to-noise ratio [28]. Nevertheless, there may be no preexisting data for many novel CRISPR screens, so BAGEL cannot be used for drug-gene interaction screens or CRISPRi/a screens.

### Permutation-based non-parametric analysis (PBNPA)

PBNPA was developed in 2017, which computed *p*-value at the gene level by the permutation test with no distribution assumptions [29]. They used median log_2_-fold changes of sgRNA counts to present overall gene effect so that it is less susceptible to outliers and off-target effects. Gene labels were randomly permuted to generate *p*-values for each gene. After that, genes with smaller *p*-values were intentionally removed and a more accurate null distribution was generated without significant genes. Updated *p*-values and FDR for each gene could also be computed from the null distribution. A common null probability distribution was employed instead of a gene-specific distribution, which will lead to more computational time. Testing with real datasets, PBNPA has better FDR control than MAGeCK, and it is also more robust to data variability.

### CRISPhieRmix

Developed in 2018, CRISPhieRmix was one of the few methods intentionally developed for CRISPRi and CRISPRa screens so far [30]. Unlike CRISPR knockout screens, the variability in CRISPRi and CRISPRa screens increases the difficulty of identifying hits. The complicated epigenetic regulation such as chromatin organization [31] and DNA folding [32] will lead to a diversified sgRNA effect. Existing methods largely relied on a hypothesis distribution while CRISPhieRmix used a hierarchical mixture model to estimate variable sgRNA efficiencies and a longer-tailed null distribution similarly with findings in gscreend [33]. Log_2_-fold changes of sgRNA were taken as input, and it was assumed that they follow a mixture distribution of effective guides and ineffective ones. FDRs were first calculated by the posterior probability that each gene is nonessential, then marginalizing all possible mixture distributions and final FDRs were obtained. Large improvements were found in CRISPRi/a screen analysis because CRISPhieRmix distinguished genes with variable guide efficiencies. However, CRISPhieRmix was largely dependent on good control guides [8] and it should be checked before the use.

### Joint analysis of CRISPR/Cas9 knockout screens (JACKS)

The problem of various sgRNA efficiencies is one of the sources of confounding in CRISPR screen analysis. Developed in 2019, JACKS is an algorithm based on Bayesian methodology that is able to model sgRNA efficiencies by obtaining information from multiple screens utilizing same sgRNA library design [34]. JACKS considered sgRNA effect as a combination of gene essentiality under different treatment and treatment-independent sgRNA efficiency. After obtaining posterior probability distributions for these factors, a gene set served as negative control for the calculation of *p*-value, which shared a similar idea in BAGEL. By better modeling the guide-specific effect in multiple screens, JACKS improves the estimate of gene essentiality compared with other methods. In this way, JACKS increased the signal-to-noise ratio and worked better especially for negative selection [28]. Although common sgRNA efficiency was assumed in JACKS, the function of gene essentiality was not. While CERES assumed that gene essentiality is common across different experiments which may be beneficial for the identification of universal hits in different cell lines, JACKS focused more on the excavation of context-specific hits.

### gscreend

Developed in 2020, gscreend focused on the accurate modelling of read count distribution in CRISPR screens for improved experiment outcomes [33]. A certain number of cells should be cultured for a powerful statistical test in CRISPR screens. For example, it is recommended that cell number should be 500 times as the number of sgRNAs in the library [5]. After comparing guide abundance through the culturing process, they found that the before/after ratios for control guides are not asymmetric in CRISPR knockout screens, which is influenced by cell proliferation and width of sgRNA abundance distribution. Thus, a skew normal distribution is used to estimate the null distribution. Gscreend cut data into different slices based on sgRNA abundance. For each interval, least quantile of squares regression is used to fit the skew normal distribution. The program calculated *p*-values for each sgRNA and aggregated sgRNA effect with α-RRA algorithm [17]. It may be useful for experiments with limited size to identify hit genes.

### Mean alterations using discrete expression (MAUDE)

MAUDE was designed for CRISPR screens with FACS readouts that sort cells into separate bins and get sgRNA abundances in each bin by NGS in 2020 [35]. When dealing with sorting-based CRISPR screens, the changes of sgRNA abundances in high expression and low expression bins will be commonly used for comparison [12]. However, it will be harder to identify hits in more than two kinds of bins. MAUDE estimated the mean effect of single guide by maximizing the likelihood of the read counts in each bin. *z*-scores can be calculated for each guide and then aggregated for the estimation of element effect, which can be either annotated by the target genes or identified by sliding window methods in tiling screens. They used Stouffer’s method to combine guide-level *z*-scores into gene-level significance. Generally, MAUDE is a useful approach for identifying regulatory elements in sorting-based screens.

## METHODS REPURPOSED FOR CRISPR SCREENS

RNA interference (RNAi) is the phenomenon of homologous mRNA degradation caused by double strand RNA (dsRNA). This approach has been used in large-scale screens to identify gene functions *in vitro* [36] and *in vivo* [37]. RNAi downregulates gene expression by mRNA degradation while CRISPR plays a role at the DNA level. Microarray experiments demonstrated that siRNA may silence numerous unintended transcripts, which will lead to off-target effects in RNAi screens [38]. RNAi may also cause the upregulation of interferon-related genes [39] and displays sequence-independent off-target effects. Compared with CRISPR screens, RNAi screens produce more systematic off-target effects [40]. Despite different mechanisms of RNAi and CRISPR Cas9 technology, the ideas of performing functional screens are similar. Thus, several computational methods originally developed for RNAi screens can also be adapted for CRISPR screen analysis.

### Redundant siRNA activity (RSA)

RSA was developed in 2007 in order to deal with the off-target effects in RNAi screens. A statistical score was designed to estimate the probability of a gene hit according to multiple siRNA effects per gene. In RSA analysis, all guides were first ranked by their signals, such as log_2_-fold change. An iterative hypergeometric distribution was then used to calculate a *p*-value, which indicated the probability of all guides targeting one gene being nonrandomly distributed at the top rankings. Guides clustered at the top were regarded as active, and the rest are labeled as negative guides. Due to the fact that RSA was probability-based, a gene with some moderately active guides was regarded to be more essential than a gene with single but extremely active guide. Due to its consideration of collective effects of all guides targeting one gene, it is also a powerful way to get rid of sgRNA off-target effects, and is used in both RNAi screens and CRISPR screens [41].

### RNAi gene enrichment ranking (RIGER)

RIGER was developed in 2008 and integrated the effects of multiple shRNAs targeting one gene to identify essential genes in RNAi screens [42]. The core of RIGER analysis was based on gene set enrichment analysis (GSEA) which utilized a weighted Kolmogorov-Smirnov (KS) statistics to test whether a predefined set of genes skewed to the top or bottom of the whole gene list [43]. RIGER considered the entire list of sgRNAs targeting the same gene due to various efficiencies of designed sgRNAs. For CRISPR screen data, RIGER first scored sgRNAs in terms of their differential effects, such as signal-to-noise ratio, between the treatment group and control group. Raw enrichment scores (ES) were then calculated in a similar manner as for GSEA analysis. Normalization was further performed to account for different numbers of sgRNAs targeting different genes by dividing ES by the mean of a null distribution generated from random permutations. RIGER offered gene rankings computed for positive scores and negative scores separately. Besides KS test, RIGER also offered a weighted sum of the first two guides (WS) and the second-best hairpin (SB) scoring methods, which depended on the selection of representative guides.

Compared with MAGeCK, which was designed for CRISPR screens analysis, RIGER had a lower sensitivity at the gene level, and it missed some of the essential genes [17]. RSA tended to have a lower specificity and reported more hit genes [17]. When the number of sgRNAs targeting one gene was decreased, both MAGeCK and RSA were robust in the identification of essential genes while RIGER seemed to be susceptible to the change of sgRNA numbers [8]. RIGER and RSA were not able to output positive selection and negative selection results simultaneously, whereas MAGeCK was able to perform bi-directional analysis.

## METHODS DESIGNED FOR SINGLE-CELL CRISPR SCREENS

It may not be accurate enough to assume a homogenous cell population when trying to analyze transcriptome profile of perturbed cells, especially in studies that diverse types of cells are involved, such as immune response or brain development. Single-cell CRISPR screens combine the advantages of CRISPR screens and scRNA-seq well. In general, the designed sgRNA library is transduced to different cell populations, which is conducive to the abundance of gene perturbations. Then, scRNA-seq serves as the readout to show how the transcriptome responds to specific perturbation, and it largely increases the number of phenotypes researchers may obtain. Thus, single-cell CRISPR screens are useful for the exploration of complicated mechanisms in heterogenous cell population. For example, researchers developed an *in vivo* perturb-seq system, introduced gene perturbation into an embryo and performed single-cell sequencing in developing brain cells to identify the functions of autism-related genes in different brain cells [44]. However, the scale of cell and gene numbers we can explore is still limited. To increase the screening power, single cell CRISPR screens can be designed to target some candidate genes in normal CRISPR screens.

Different technologies have also been developed to perform single-cell CRISPR screens. In Perturb-seq [14] and CRISP-seq [15] platforms, guide RNAs are identified with its transcriptome through a barcode whose expression is regulated by Pol II. Guide barcode (GBC) PCR data is required to pair gene perturbations with cell barcodes. Thus, the swapping of sgRNA-barcode relationship because of virus template switching is a big concern [45]. However, in CROP-seq [16], one copy of the guide is designed to be transcribed under Pol II regulation, which can be sequenced directly and requires no pairing process. A limitation of CROP-seq is the low identification rate at about 50% of sgRNAs from scRNA-seq data [16]. Further modified vector by positioning the barcode in the 3′UTR of antibiotics resistance gene enabled barcode identification by poly(A) capturing process in scRNA-seq, which largely increases the successful rate of identifying sgRNA-cell association [45].

It is very challenging to analyze single cell CRISPR screens because of large scale and high variation. Gene expression clustering using similar mechanism in scRNA-seq analysis can be used. Each cell in the screen is usually categorized into different clusters by clustering analysis on their transcriptome [46]. Then, researchers are able to determine if a particular set of sgRNAs is enriched in one cluster by chi-squared or hypergeometric distributions. Meanwhile, there are some single cell sequencing analysis methods specially designed to adapt to the characteristics of CRISPR screens.

### Multi-input-multi-output single cell analysis (MIMOSCA)

MIMOSCA is the analysis method designed for Perturb-seq in 2016 [14]. The inputs include an expression matrix output of high-throughput scRNA-seq. In order to pair guide barcodes (GBC) with perturbations [47], GBC PCR data and a database for pre-associated sgRNA-GBC pairs are required. Unlike other methods, sgRNA-target information or negative controls is not used in MIMOSCA [48]. They used a linear regression between the expression matrix and the design matrix, and they fit the coefficient matrix with elastic net regularization. A permutation-based test is used to further estimate the significance of coefficients obtained from the matrix. Cell state classifications on wild type or control cells can also be used as covariates in the model instead of gene expression. Under this linear regression framework, nonlinear interactions can also be incorporated by introducing interaction between covariates such as genetic interactions. Nevertheless, the reverse transcriptase may move from one template to another when two virus genomes are non-covalently linked during lentiviral package, which will lead to barcode recombination and abate the accuracy [47].

### Model-based understanding of single-cell CRISPR screening (MUSIC)

Developed in 2019, MUSIC [49] is an integrated workflow for single-cell CRISPR screen analysis. A machine learning methodology called Topic Modeling [50] was used for the analysis, which is able to cluster word groups for the best characterization of documents. As an analogy, single cells with gene perturbations are regarded as documents while gene expression is taken as words in the documents. MUSIC first assessed the quality of the data such as cell quality, sgRNA knockout efficiency and cell number per perturbation, through which cells with ineffective editing will be filtered. Then, the topic model framework is used to capture functional topics of perturbed cells. Finally, MUSIC estimates the single perturbation effect on whole gene expression profiles. In this way, MUSIC is capable of detecting a phenotype with a group of differentially expressed genes. Compared with other methods, MUSIC can detect subtle changes in gene expression profile and can be applied to different single cell CRISPR screen platforms.

### scMAGeCK

Single-cell MAGeCK [48] was developed from the previous MAGeCK [17] models in 2020 and contained two models: RRA and LR. scMAGeCK-RRA was able to identify perturbations affecting the expression of one gene. Single cells were first ranked based on the target gene expression, and RRA was used to test if cells with particular perturbations are enriched at the top or bottom of the list. The whole process is very similar to MAGeCK methodology. As a non-parametric test, scMAGeCK-RRA was able to detect non-linear expression relationship. Another section, scMAGeCK-LR, was a linear regression model for examining all gene perturbations’ effect on the whole transcriptome. Using the matrices indicating gene expression in different cells and the identity of sgRNAs in the cell, the effect of gene perturbation is estimated with ridge regression, where positive scores indicate a positively selected gene and vice versa. However, scMAGeCK only supports data obtained from CROP-seq [16], in which sgRNA itself serves as a barcode. The limitation of scMAGeCK is that it is not suitable for other sequencing methods that need to determine sgRNA-barcode association, and that sgRNAs are identified from scRNA-seq with limited sensitivity [45].

### Mixscape

Expanded CRISPR-compatible cellular indexing of transcriptomes and epitopes by sequencing (ECCITE-seq) [51] is a technology that is developed from previous CITE-seq [52] and facilitates simultaneous detection of epitopes and transcriptomes in single-cells in the context of pooled CRISPR screens. As part of the open-source R package Seurat, Mixscape was designed for the analysis of ECCITE-seq data. This idea was inspired by a classification model mixture discriminant analysis (MDA) with the assumption that individual cells with certain gene perturbation may fall into different groups and each group includes perturbed cells and non-perturbed cells similar to control cells. Gaussian distributions representing knockout cells and non-perturbed cells were then used to model the mixture of population. By finding cells escaping gene perturbation, Mixscape increased the signal-to-noise ratio in single-cell CRISPR screen analysis. Benchmarked against MIMOSCA [14] and MUSIC [49], Mixscape is able to identify perturbed cells with high sensitivity. However, Mixscape relied on the detection of transcriptomic changes to classify cell populations, which may be not able to identify perturbations causing epigenetic changes and protein level changes. Moreover, the binary discrimination of perturbed and non-perturbed cells may be oversimplified because CRISPR/Cas9 technology causes diverse sequence variants.

### Single-CEll PerTurbation screens via conditional REsampling (SCEPTRE)

SCEPTRE [53] is derived from the conditional randomization test, and independent on the accurate specification of the expression model. A resampling methodology is utilized to account for heterogeneity in different cells. SCEPTRE first fit logistic regression of sgRNA on some technical factors to obtain fitted probabilities. The null distribution is generated for a gene and sgRNA by reassigning sgRNA for each cell in terms of individual probability of perturbation. An improved negative binomial regression is used to estimate sgRNA effects, which includes sequencing depth as a covariate. SCEPTRE can deal with technical confounders better than other methods. To accelerate the program, the resampled *z*-values are calculated from a skew-t distribution similar to CRISPhieRmix [30]. Finally, a *p*-value is computed by comparing *z*-values to the generated null distribution.

## METHODS DESIGNED FOR DRUG-GENE INTERACTION SCREENS

### MAGeCK-MLE

Embedded in MAGeCK-VISPR workflow, MAGeCK-MLE algorithm [21] is able to analyze complicated experiment conditions such as different time points or cell lines. Unlike MAGeCK, the negative binomial distribution is determined by sequencing depth, guide efficiency and drug treatments in MLE. Beta-scores are estimated by maximizing the likelihood of fitting all guide read counts on all samples. A positive score indicates positive selection whereas a negative one is the symbol of negative selection. The significance of β score can be estimated either by permutation or Wald test. MAGeCK-MLE is able to handle the pair samples at each time point in CRISPR screens for drug resistance. Nevertheless, the EM algorithm is based on an iterative process, which will make it work slower than other algorithms. Moreover, MAGeCK-MLE can incorporate sgRNA efficiency information calculated based on guide sequence from spacer scoring for CRISPR (SSC) [54].

### DrugZ

Based on the framework of CRISPR screens, drug treatment is added to the cell population which enables researchers explore the mechanism for drug resistance. Unlike essential gene screen analysis where the read counts of sgRNAs after culturing for a period of time are compared to the initial sample, the abundance of sgRNA in a drug-treated group is compared to an untreated group at each time point as a pair in drug-gene interaction screens. DrugZ is an algorithm intended to identify synergistic and suppressor interactions between chemical compounds and genes from CRISPR screens [55]. The log_2_ fold changes of sgRNAs are calculated after normalizing the read count with the control group at each time point. A *z*-score will be calculated for each guide, and the variance will be estimated by empirical Bayes. Gene-level *z*-scores are obtained by combining guide-level scores, after which we can get *p*-values from normal distribution. Both of synergistic and suppressive interactions can be discovered in one experiment at the same time. DrugZ works well with CRISPRko, CRISPRi/a screens and it has a higher sensitivity than other algorithms.

## CONCLUSION AND DISCUSSION

In summary, the most challenging part for CRISPR screen analysis is to estimate sgRNA abundance and to aggregate sgRNA effects with the same target to infer gene-level effect. Different methods have different hypothesis, and different distributions are utilized such as normal distribution, Poisson distribution and negative binomial distribution. Negative binomial model is more suitable because it considers the large variance of read counts in NGS data [17] which effectively reduces false positives. Meanwhile, other methods circumvent the estimate of sgRNA distribution. For example, PBNPA [29] relies on non-parametric permutation of gene labels and BAGEL [26] is derived from Bayes’ theorem so that previously published essential and nonessential gene sets are used. To aggregate sgRNA effect, a variety of tests are introduced to the algorithm such as RRA [17], KS test [42] and MLE [21]. Experiment design and analysis methodology also work in an interdependent way. If we have a thorough understanding of the phenotype, we are able to design a proper group of control guides so that the *t*-test will work well [8]. To get robust results, sometimes several methods with different mechanisms were used together, and overlapped hit genes can be considered as top candidates [56]. Unlike other methods that can only analyze a single screen each time, MAGeCK-MLE, CERES and JACKS are suitable for multiple screen designs, and they can be used if researchers are using multiple cell lines or want to identify cell type-specific hits.

Copy number variation (CNV) is also a significant phenomenon in genetic variation [57] that should be considered in CRISPR screen analysis because copy number alterations commonly happen in cancer cells utilized in CRISPR screens. In CRISPRko screens, double-strand breaks in gene regions with a high copy number may lead to false positives. In CRISPRa/i screens, the copy number variation of lncRNAs [58] may also lead to different degrees of activation or inhibition of lncRNA transcription. CRIS.py analysis can be used to screen cell population derived from a single cell, and it is able to predict the copy number of alleles in each cell population by measuring the ratio of indels [59]. CERES uses log_2_-fold change of sgRNA read counts between control and treatment group, and computes it as a sum of knockout effect and copy number effect, which is determined by the targeted loci and the copy number at each locus with the input of CNV file [60]. Additionally, a CNV function is incorporated in MAGeCK which requires no input of available CNV file because MAGeCK utilizes a sliding-window method for the estimate of CNV in various cell lines and samples [17].

Some platforms such as CRISPRCloud [61], CRISPRAnalyzeR [62] and Platform-independent Analysis of Pooled Screens using Python (PinAPL-Py) [63] offer researchers web-based interactive analysis of CRISPR screens. In addition to gene ranks, these integrated workflows contain quality control, visualization and downstream analysis modules, which enables wet lab researchers to analyze CRISPR screens by themselves without much computational skill. Although the operation becomes simple, it is still necessary to understand the mechanism of CRISPR screen analysis in order to better understand the results and deal with problems. On the other hand, we may need robust methods when we deal with novel phenotypes. In general, MAGeCK is the state-of art method nowadays that is suitable for various screens, and it is vigorously developed into different workflows. There are also methods with the focus of a particular type of screens, such as CRISPhieRmix [30] for CRISPRi/a screens, MAUDE [35] for sort-based screens, drugZ [55] for drug resistance screens and scMAGeCK [48] for single-cell CRISPR screens.

Although single-cell CRISPR screen is a promising tool to uncover complicated interactions, the number of cells that can be sequenced and analyzed in a screen is still limited. The identification of barcodes in single cells also requires improvement of the sensitivity of scRNA-seq and higher pairing accuracy. The cost of time and money for single-cell CRISPR screens may be further reduced by specifically amplification of target genes or depletion of unrelated genes with high abundance. Some algorithms that can analyze single-cell CRISPR screen have been developed, but they are often designed for a specific methodology of building a library and lack of generality. In addition to the false positives or false negatives problems similar to traditional CRISPR screen analysis, it is even more challenging to accurately model hierarchies in different signaling pathways, which requires development of novel algorithms to deal with a huge amount of data with intrinsic noise. Moreover, phenotype changes may be not restricted to transcriptomic level, and efforts should be made to further incorporates diverse phenotypes such as chromatin state [64] and protein expression [51].

Data sharing plays an important role in genomic discovery. In order to make it easier for researchers to compare the experimental results in parallel, some CRISPR screen databases have been developed. CRISP-view [65] is a comprehensive database of processed CRISPR screen data by MAGeCK-VISPR pipeline with quality control, which is developed by the same group of MAGeCK. It is a web-based tool for searching and visualizing sgRNA expression across different datasets. Although CRISPR screens *in vivo* and *in vitro* have enabled the discovery of many hit genes, to perform large-scale CRISPR screens of real patient for target identification is not feasible yet. Recently, by integrating shRNA screens, CRISPR/Cas9 screens, transcriptomics and mutation profiles of TCGA samples, a deep learning method is able to predict cancer-specific vulnerabilities in clinical samples with *in silico* CRISPR/RNAi screens [66]. This study indicates that the further integration of CRISPR-screening, tumor sample, and clinical data may guide us to better discover therapeutic targets.

## Figures and Tables

**Table 1:** Tools for analyzing CRISPR screen data

	Tool	Year	sgRNA rank	Gene rank	FDR	QC	Visualization	Latest version	Total citations
Methods repurposed for	RSA	2007	Hypergeometric distribution	Ranking-based statistics	N	N	N	1.9 (Jan. 2019)	314
CRISPR screens	RIGER	2008	Signal-to-noise ratio	Kolmogorov-Smirnov test	N	N	N	2.0.2 (Apr. 2018)	541
General methods for CRISPR screens	MAGeCK (RRA)	2014	Negative binomial distribution	Robust rank aggregation	Y	Y	Y	0.5.9 (Jul. 2019)	794
	HiTSelect	2015	Poisson distribution	Stochastic multiobjective ranking	Y	N	Y	First released in Jul. 2014	56
	BAGEL	2016	Reference gene set distribution	Bayes factor	Y	N	N	2.0 (Aug. 2020)	130
	ScreenBEAM	2016	Normal distribution	Bayesian hierarchical modeling	Y	N	N	1.0 (Jul. 2016)	34
	ENCoRE	2017	Normal distribution	Student’s *t*-test	Y	N	Y	First released in 2017	14
	PBNPA	2017	NA	Non-parametric permutation	Y	N	N	0.0.3 (May 2018)	19
	PinAPL-Py	2017	Negative binomial distribution	α-RRA, STARS	Y	Y	web-based	NA	33
	CRISPRAnalyzeR	2017	DESeq2 (based on gene-level read counts), MAGeCK, sgRSEA, edgeR, BAGEL, ScreenBEAM, Mann-Whitney Test	Eight approaches	Y	Y	web-based	1.50 (Jan. 2018)	NA
	CRISPhieRmix	2018	Hierarchical mixture model	Expectation maximization algorithm	Y	N	N	1.1 (Apr. 2019)	14
	JACKS	2019	NA	Bayesian hierarchical modeling	Y	N	N	Updated in Oct. 2020	30
	CRISPRCloud2	2019	Beta binomial distribution	Fisher’s test	Y	Y	web-based	NA	16
	gscreend	2020	Skew-normal distribution	α-RRA	Y	Y	N	1.0 (Mar. 2020)	8
	MAUDE	2020	Maximum likelihood estimate	Stouffer’s *z*-method	Y	N	N	First released in 2020	4
Methods for CRISPR chemogenetic	MAGeCK-VISPR (MLE)	2015	Negative binomial distribution	Maximum likelihood estimation	Y	Y	Y	0.5.6 (Dec. 2020)	173
screens	DrugZ	2019	Normal distribution	Sum *z*-score	Y	N	N	First released in 2019	29
Methods for single-cell	MIMOSCA (Pertrub-seq)	2016	NA	Linear model	Y	N	N	Updated in Aug. 2019	672
CRISPR screens	MUSIC	2019	NA	Topic model	N	N	N	Updated in 2020	22
	scMAGeCK (CROP-seq)	2020	NA	RRA/LR	Y	N	N	First released in 2020	13
	SCEPTRE	2020	Negative binomial regression	Skew-t distribution	Y	N	N	First released in 2020	NA
